# Bypass surgery in a Jehovah’s Witness with critical limb ischemia and end stage renal disease: a case report

**DOI:** 10.1093/jscr/rjaf795

**Published:** 2025-10-09

**Authors:** Eol Choi

**Affiliations:** Division of Vascular Surgery, Department of Surgery, Soonchunhyang University College of Medicine and Soonchunhyang University Bucheon Hospital, 170, Jomaru-ro, Bucheon-si, Gyeonggi-do 14584, Republic of Korea

**Keywords:** critical limb ischemia, blood transfusion/refusal, renal dialysis

## Abstract

A 60-year-old female Jehovah’s Witness with critical limb ischemia and end-stage renal disease underwent staged revascularization including endovascular intervention, free flap reconstruction, and ultimately femoropopliteal bypass using in situ great saphenous vein. Despite maximal blood conservation strategies—erythropoiesis-stimulating agents, intravenous iron, and albumin—her postoperative hemoglobin dropped to 4.7 g/dl without any evidence of active bleeding. She declined allogeneic transfusion and developed multiorgan failure, resulting in death on postoperative Day 3. This case highlights the clinical and ethical complexities of managing high-risk vascular patients who refuse blood transfusion, emphasizing the need for individualized risk assessment, careful perioperative planning, and consideration of patient autonomy under potential external influence.

## Introduction

Critical limb ischemia (CLI) represents the most advanced form of peripheral arterial disease and carries high risks of limb loss and mortality, especially in patients with multiple comorbidities such as diabetes mellitus and end-stage renal disease (ESRD) [1]. These patients often present with poor wound healing, calcified vessels, and limited options for revascularization. Although endovascular techniques have become increasingly favored for limb salvage due to their minimally invasive nature, their long-term success in heavily calcified arteries remains suboptimal [2].

In such cases, surgical bypass remains the most durable option, albeit with increased perioperative risks. Management becomes significantly more challenging in Jehovah’s Witness patients, who refuse blood transfusion based on religious beliefs [3]. Despite recent reports of successful outcomes in transfusion-free major surgeries using blood conservation strategies, profound anemia remains a life-threatening complication, particularly in patients undergoing complex vascular procedures [4].

Here, we present a case of a Jehovah’s Witness patient with CLI and ESRD who underwent multiple staged revascularization procedures. Despite successful technical outcomes and the use of aggressive blood conservation strategies, the patient developed profound anemia, ultimately resulting in multiorgan failure and death.

## Case report

**Figure 1:**
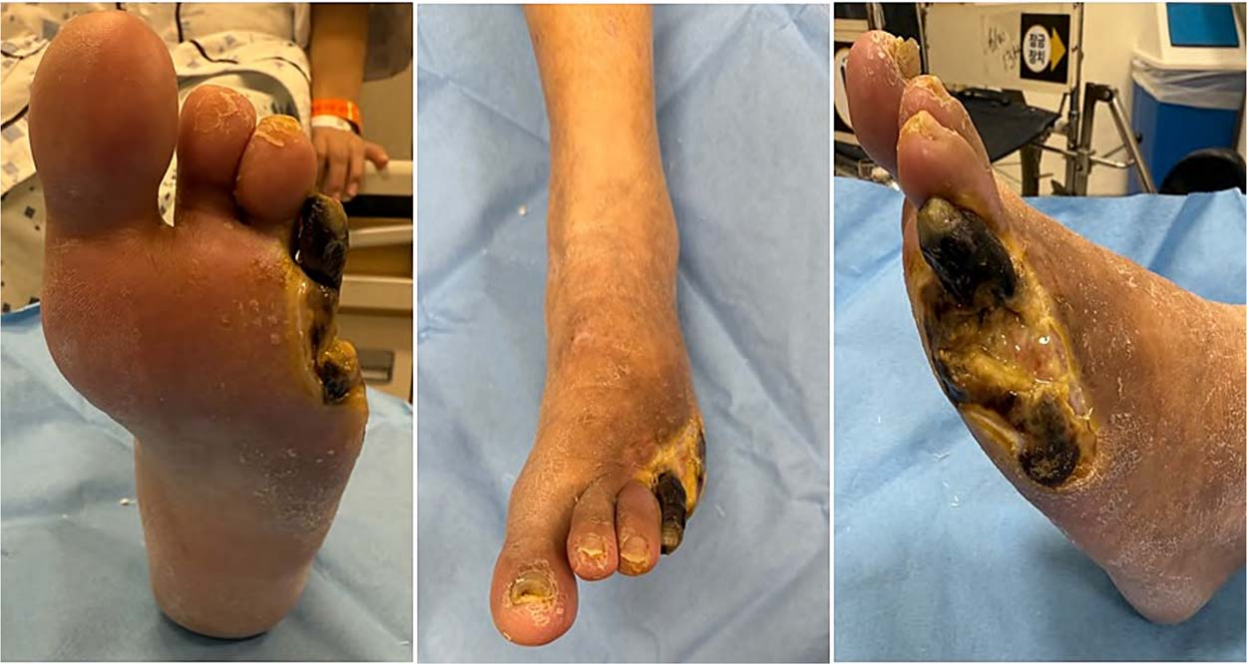
Clinical photograph of the left foot on initial presentation at our institution.

A 60-year-old female Jehovah’s Witness with diabetes and ESRD on hemodialysis presented with chronic non-healing forefoot ulcers and dry gangrene. She had previously undergone transmetatarsal amputation of the fifth toe, but necrosis progressed. Initial percutaneous transluminal angioplasty of the superficial femoral artery (SFA) failed due to severe calcification. A second attempt via retrograde posterior tibial artery access and PIERCE technique was successful, leading to partial wound improvement (Fig. 1).

**Figure 2:**
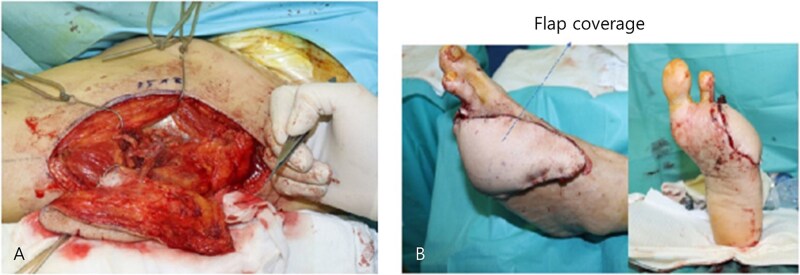
Intraoperative photographs of the ALT free flap procedure. (A) Donor site on the anterolateral thigh after flap harvest, showing the surgical field prior to closure. (B) Immediate postoperative view of the left foot following successful inset of the ALT free flap, with microvascular end-to-end anastomosis to the dorsalis pedis artery.

**Figure 3:**
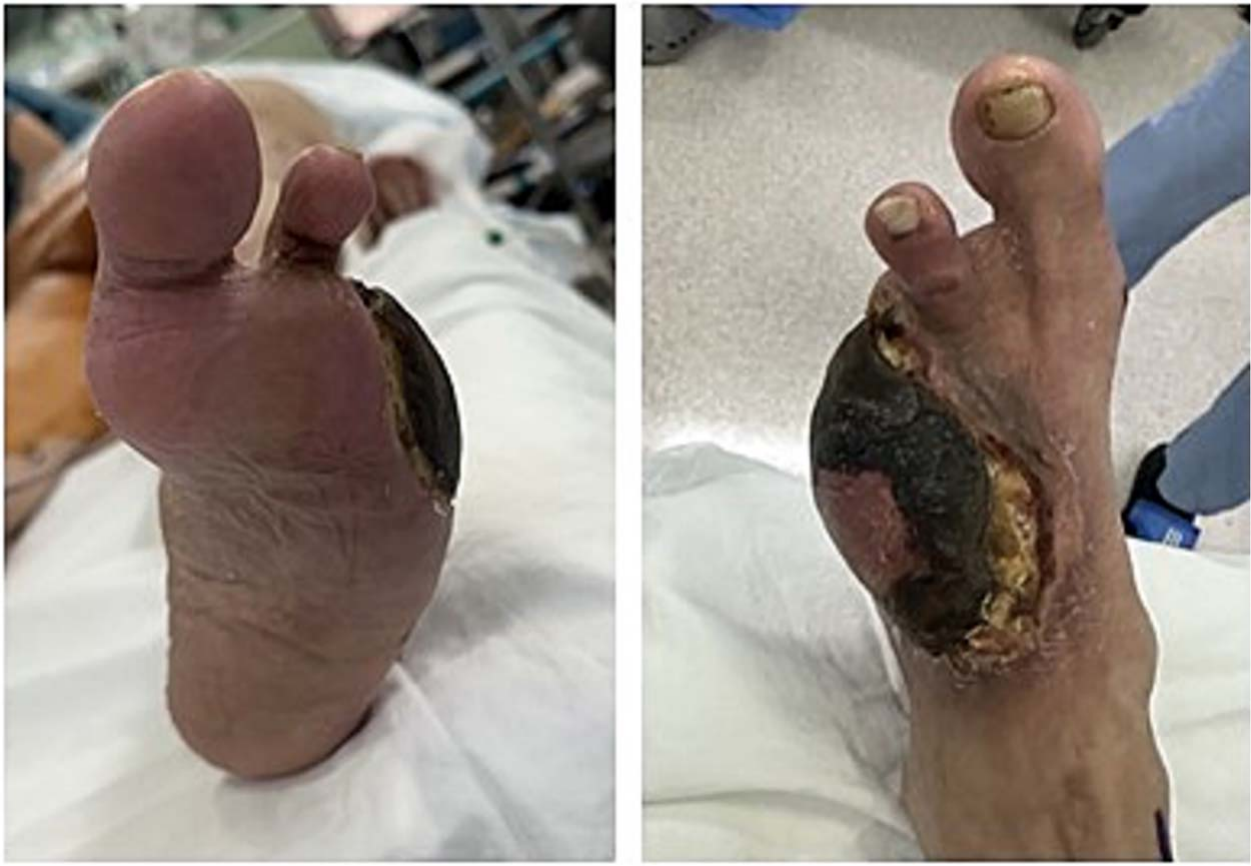
Clinical photograph of the left foot upon readmission three months after free flap reconstruction.

**Figure 4:**
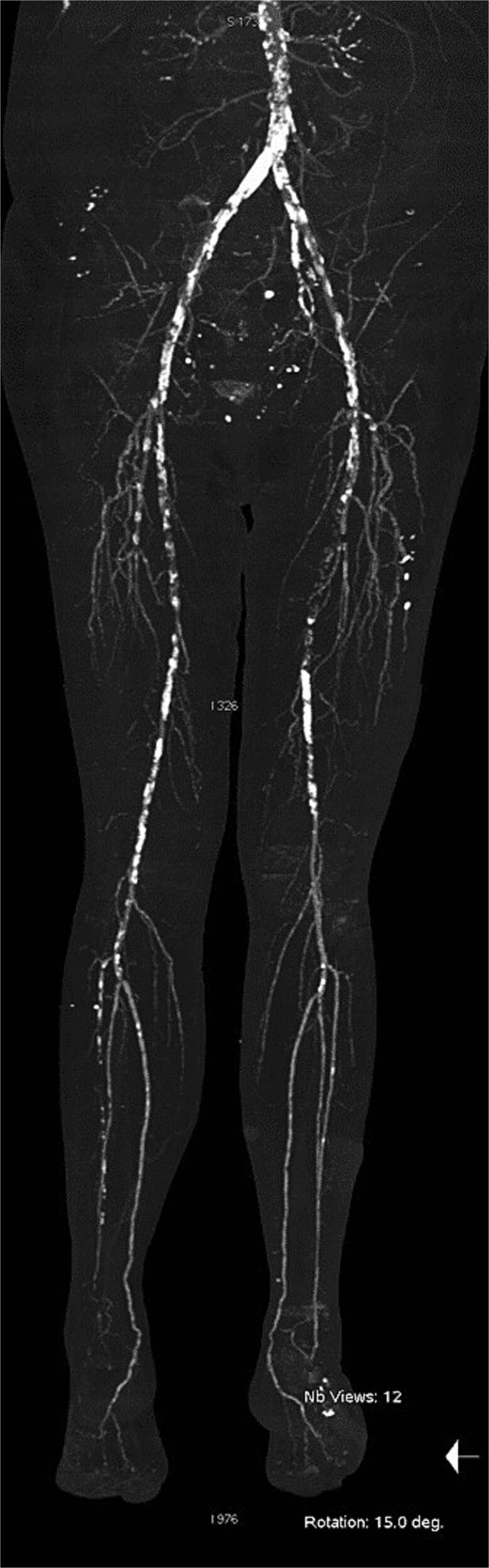
Computed tomography angiography at the time of readmission.

At our center, the patient underwent third to fifth toe ray amputation and subsequent anterolateral thigh free flap for soft tissue coverage (Fig. 2). The flap was initially viable, but partial necrosis developed after 3 months (Fig. 3). CT angiography revealed SFA re-occlusion (Fig. 4). Considering the limited durability of prior interventions, surgical bypass was performed. Imaging showed severe calcification of the external iliac, common femoral, and proximal SFA. She underwent femoral endarterectomy and in situ great saphenous vein bypass to the below-knee popliteal artery. Completion angiography confirmed good graft flow.

The operation lasted 6 h with 300 ml blood loss. Her hemoglobin dropped from 9.9 to 7.0 g/dl postoperatively. Erythropoietin, IV iron, and albumin were administered immediately. On postoperative day (POD) 1, her Hb fell further to 5.7 g/dl without signs of bleeding. Transfusion was recommended, but both patient and guardian refused due to religious beliefs.

On POD 2, she became hypotensive, requiring vasopressors and continuous renal replacement therapy. By POD 3, she showed progressive organ failure with hyperkalemia, acidosis, liver enzyme elevation (>2000 IU/l), and troponin *I* > 10 000 ng/l. Despite intensive support, including vasopressin, corticosteroids, and sedation, she died later that day from multiorgan failure related to anemia.

## Discussion

CLI in patients with ESRD and diabetes is difficult to manage, particularly when arterial calcification limits endovascular options. Surgical bypass is often required for durable perfusion but comes with greater perioperative risk. In Jehovah’s Witness patients, the refusal of transfusion further complicates care. Although blood conservation techniques can support hematologic recovery, they may be insufficient in prolonged or complex surgeries [5].

Preoperatively, correction of anemia with erythropoiesis-stimulating agents, intravenous iron, and nutritional support is essential. Albumin may be used to maintain oncotic pressure and avoid hemodilution [6, 7]. Intraoperative strategies include minimal-access techniques, meticulous hemostasis with electrocautery and topical agents, and consideration of cell salvage if acceptable [8, 9]. Anesthesiologists play a key role in maintaining normothermia and optimizing fluid balance [10].

In our case, the option of primary amputation was considered within the multidisciplinary team. However, infrainguinal bypass is generally associated with relatively modest intraoperative blood loss and an acceptable operative risk profile. Johnson *et al*. reported that transfusion was not universally required even in infrainguinal bypass patients with lower preoperative hemoglobin levels, suggesting that our patient, with a hemoglobin of 9.9 g/dl, was not at high anticipated risk for transfusion [11]. Given these considerations and the patient’s strong preference for limb salvage, we elected to proceed with bypass rather than primary amputation.

While some reports describe survival after surgery with hemoglobin levels as low as 1.8 g/dl, such cases are rare and rely on exceptional physiologic compensation [12]. In high-risk patients with multiple comorbidities, such as CLI and ESRD, even aggressive blood conservation may not be sufficient [13]. Therefore, transfusion-free surgical planning must be individualized, with a clear understanding of the patient’s physiological reserve and expected surgical burden.

This case also raises questions about autonomy. The patient’s family reportedly received financial support from their religious community, which may have influenced decision-making. Autonomy assumes freedom from coercion, yet in real-world contexts, such as religious or financial dependency, true independence is difficult to confirm [14]. Medical teams should provide opportunities for private, unbiased counseling to ensure that patients’ choices are genuinely their own.

## Conclusion

Successful surgery does not guarantee survival when transfusion is not an option. Jehovah’s Witness patients undergoing high-risk procedures require a multidisciplinary approach, aggressive blood conservation, and transparent discussions about outcomes. Ethical issues surrounding autonomy should be actively addressed, particularly when external influences may affect life-or-death decisions. Preoperative planning must assess not only technical feasibility but also the patient’s capacity to physiologically withstand expected anemia.
